# Thyroid “claw sign” a useful diagnostic marker in the outsized lesions of isthmus: A large colloid cyst.

**DOI:** 10.1016/j.radcr.2021.04.012

**Published:** 2021-04-30

**Authors:** Adeena Khan, Mamoona Sultan, Waleed Fawzy, Syed Shahid Habib, Muhammad Usman Ul Haq

**Affiliations:** a Department of Radiology and Medical Imaging, King Saud University Riyadh 11451, Saudi Arabia; b Department of Internal Medicine, King Saud University, Riyadh, Saudi Arabia; c Department of Clinical Physiology, King Saud University, Riyadh, Saudi Arabia; d Department of Orthopedics, Jarir Medical Centre, Riyadh, Saudi Arabia

**Keywords:** Isthmus, Colloid cyst, Comet tail artifact, Claw sign, Computed tomography (CT)

## Abstract

Thyroid isthmus lesions are generally small sized and can be solid or cystic. Discerning isthmic origin of a large nodule, especially if purely cystic, can become a diagnostic challenge because of thin thyroid tissue in it. We report a case of a 68-year-old male patient who had 6 weeks history of non- inflammatory central neck swelling associated with recent dysphagia, for which he underwent ultrasound and computed tomography (CT) scan examinations. Colloid nodules usually do not require further attention. Despite being commonest and benign thyroid nodules, they may require treatment if causing pressure symptoms. Its imaging characteristics can be variable, but they usually exhibit comet tail artifacts on ultrasound. In equivocal cases, claw sign on CT scan is diagnostic to confirm the site. Radiologists have a principle role to rule out other differentials of cystic neck lesions by careful examination of imaging features. In our case, CT scan allowed to rule out primary differential of thyroglossal cyst and guided clinicians for specific management plan.

## Introduction

Pure cystic thyroid lesions are considered almost always benign but their diagnosis and treatment are sometime very important especially when having atypical morphology and location as in our patient.

About 15%-50% of solitary thyroid nodules are cystic [1,2]. Colloid nodules having cystic component are termed as colloid cysts (CC) and these nodules are the commonest thyroid nodules [3], [4]**–**5].

The sequence of formation of these nodules is still debatable but is proposed to be due to imbalance in the steps of thyroid hormone formation and regulation [6].

Although, it is mostly asymptomatic but has potential to be a cause of presenting symptoms due to its size, location and intracystic secondary complications. Its management recommendation is neither follow up nor surgical intervention but occasionally its presenting symptoms set it out for aspiration. Radiologist plays primary role in its diagnosis and introduction of other differentials by assessing some important imaging signs [7].

Our case highlights that isthmic CC can also compel us to ponder for differentials of other cystic thyroidal and extrathyroidal lesions necessitating cross-sectional imaging, where claw sign can help us to narrow our differentials and guide targeted management. Description of this report can help radiologists in making sonographic impression in their mind that CC does not always has comet tail artifacts of colloid crystals and lobar location.

## Case report

A 68-year-old male presented to the head and neck clinic with history of a progressive midline lower neck swelling which appeared suddenly, increased in size progressively and became prominent over 6 weeks. On examination, the swelling was about 5 × 2 cm in size, mobile and not adherent to the skin or underlying structures. The swelling was not prominent on tongue protuberance. Thyroid was impalpable and patient was clinically euthyroid with no evident Grave's disease features. It was neither associated with fever nor any other constitutional symptoms. His pressure symptom includes mild dysphagia to solids which compelled him to visit our hospital, but he did not encounter any respiratory complaint.

Patient was not on any anticoagulants, but he was taking regular medications for diabetes and hypertension for many years.

**Fig. 1:**
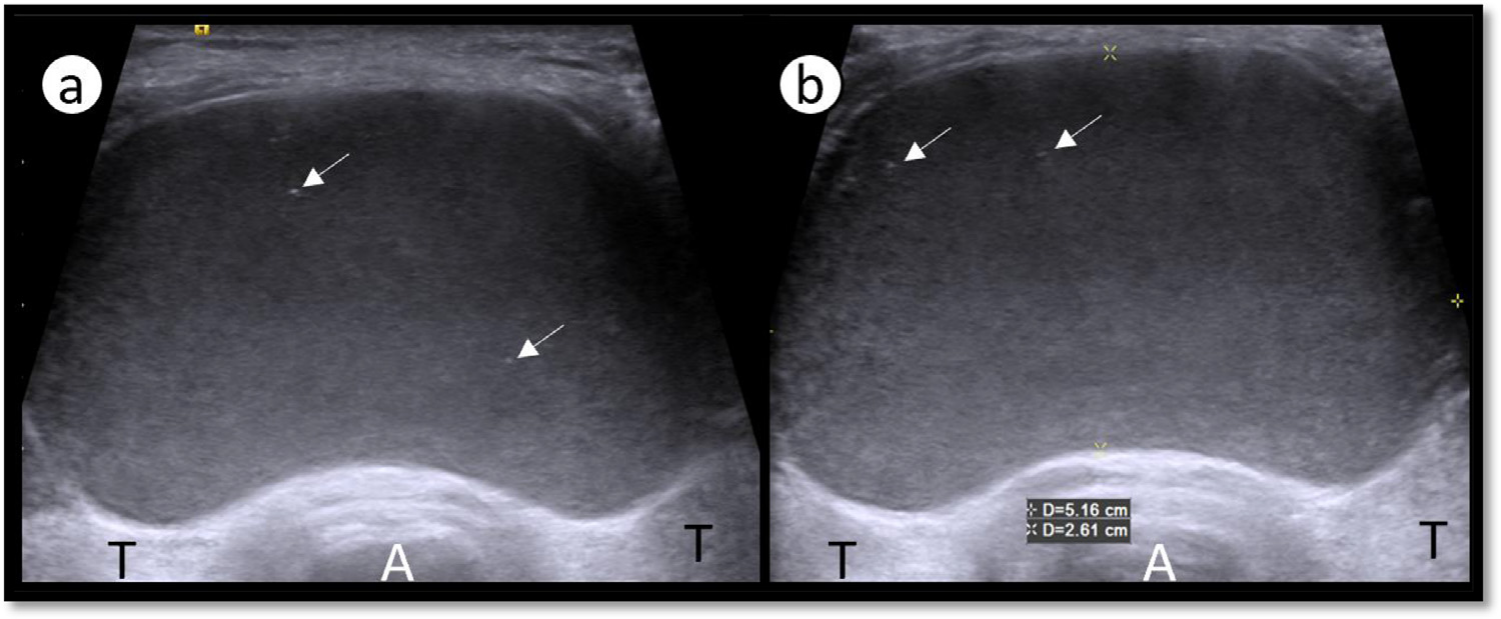
(a,b): Ultrasound neck showing a large cyst having homogenously low echogenic content with few tiny echogenic foci showing no comet tail artifact (arrows). Thyroid lobes (T) and airway (A).

**Fig. 2:**
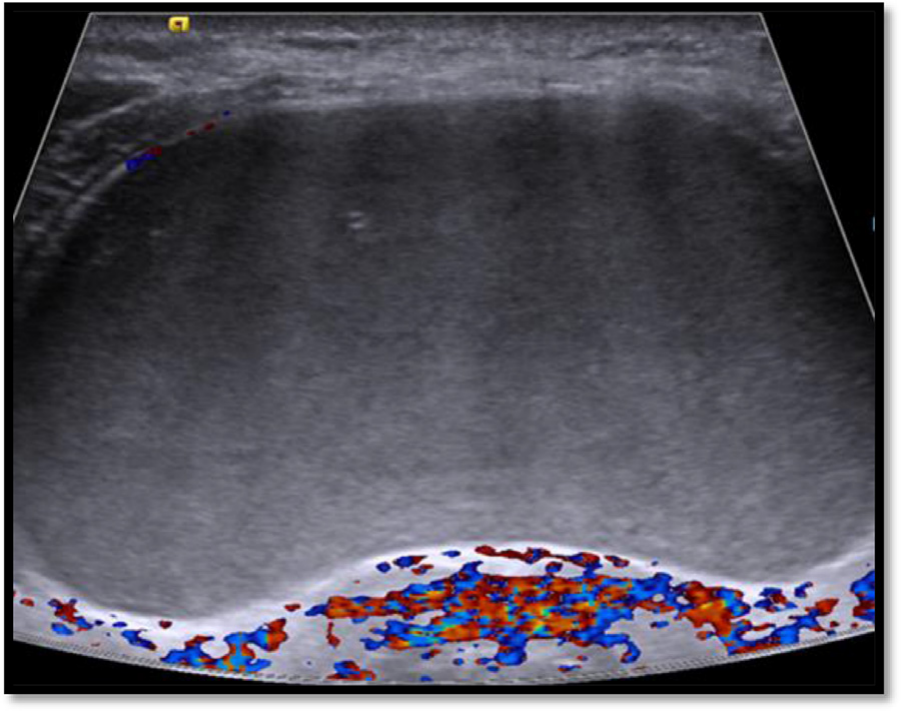
Doppler ultrasound showing avascular cyst.

**Fig. 3:**
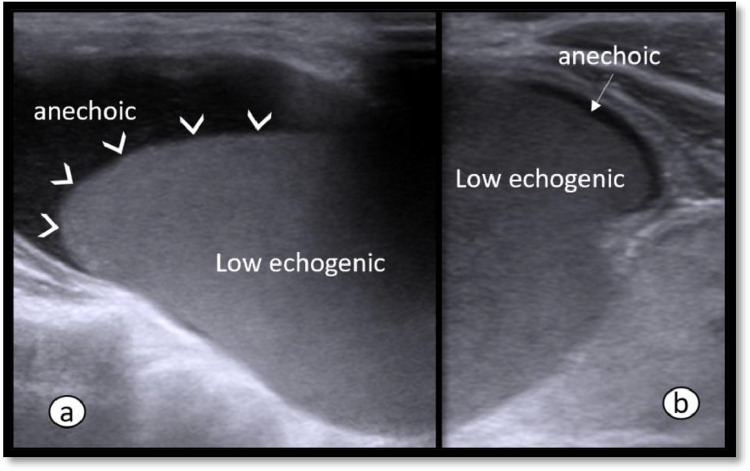
(a,b): Ultrasound showing internal content of the colloid cyst. Basal homogenous low echogenic and upper anechoic content(a,b). Arrow heads are showing curved upper margin of the basal content (a).

For treatment decision, the patient was referred to radiology department. Ultrasound neck showed a large well defined thin-walled cystic neck lesion measuring about 5.1 × 2.6 cm (transverse x anteroposterior). There was no appreciable internal septation or solid tissue in it (Fig. 1). No hypervascularity was noted within or periphery of the lesion (Fig. 2). The internal content of the cyst was of 2 types, basal part was homogenously low echogenic with curved upper border while rest of the part was filled up with anechoic fluid (Fig. 3). Few tiny echogenic foci were also seen in its low echogenic part which were not giving any comet tail artifact to be confidently called as colloid content (Fig. 1). Thyroid gland was not easily visible due to lateral push by this large cyst. Thyroid isthmus was hardly visible clearly. Lobes were found to be normal, but their echotexture was heterogenous and micronodular with normal vascularity and intact echogenicity. According to the location and content of cyst, the closest sonographic differential in our patient was thyroglossal cyst, but it is usually smaller in size, moves with tongue protrusion and more cranial in position. Other differentials could be hemorrhagic or fourth branchial cleft cyst.

**Fig. 4:**
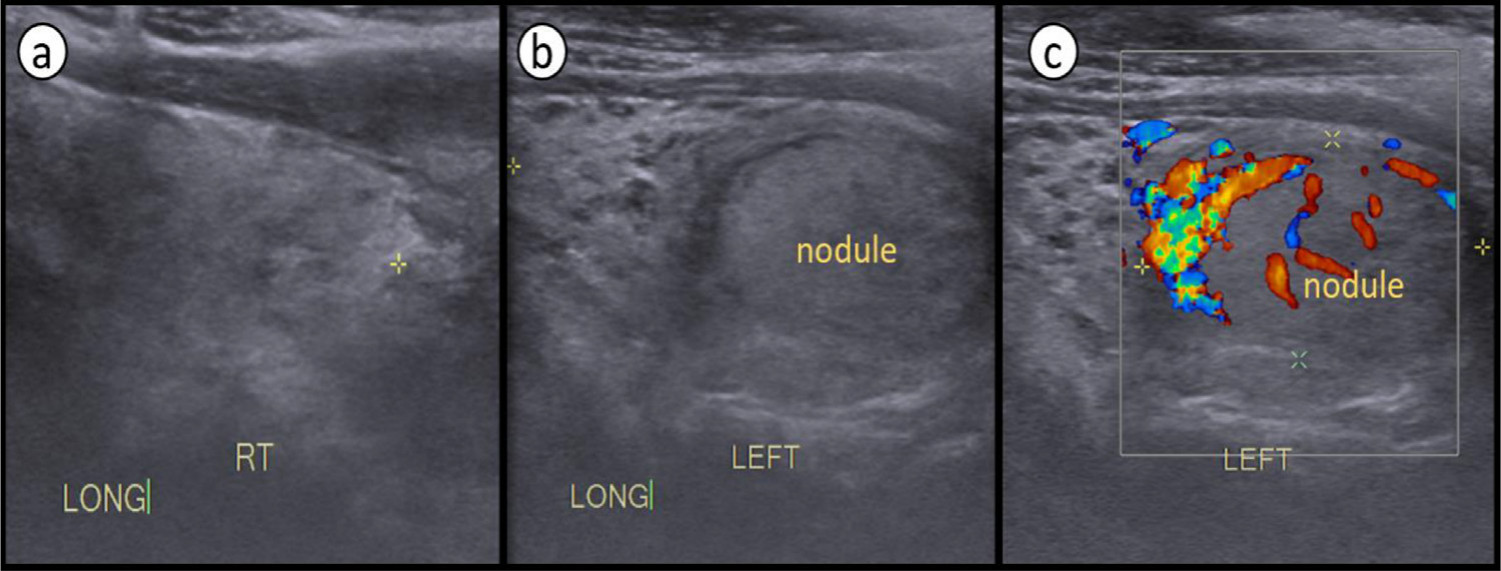
(a-c): Longitudinal ultrasound images of the thyroid lobes. Both lobes showing heterogenous echotexture (a-c). Solitary solid nodule in the left lobe (b,c,C), showing vascularity on doppler ultrasound (c).

Left lobe was having a solid well-defined isoechoic nodule measuring around 2.5 × 2.0 × 1.5 cm with some internal vascularity on doppler ultrasound (Fig. 4).

**Fig. 5:**
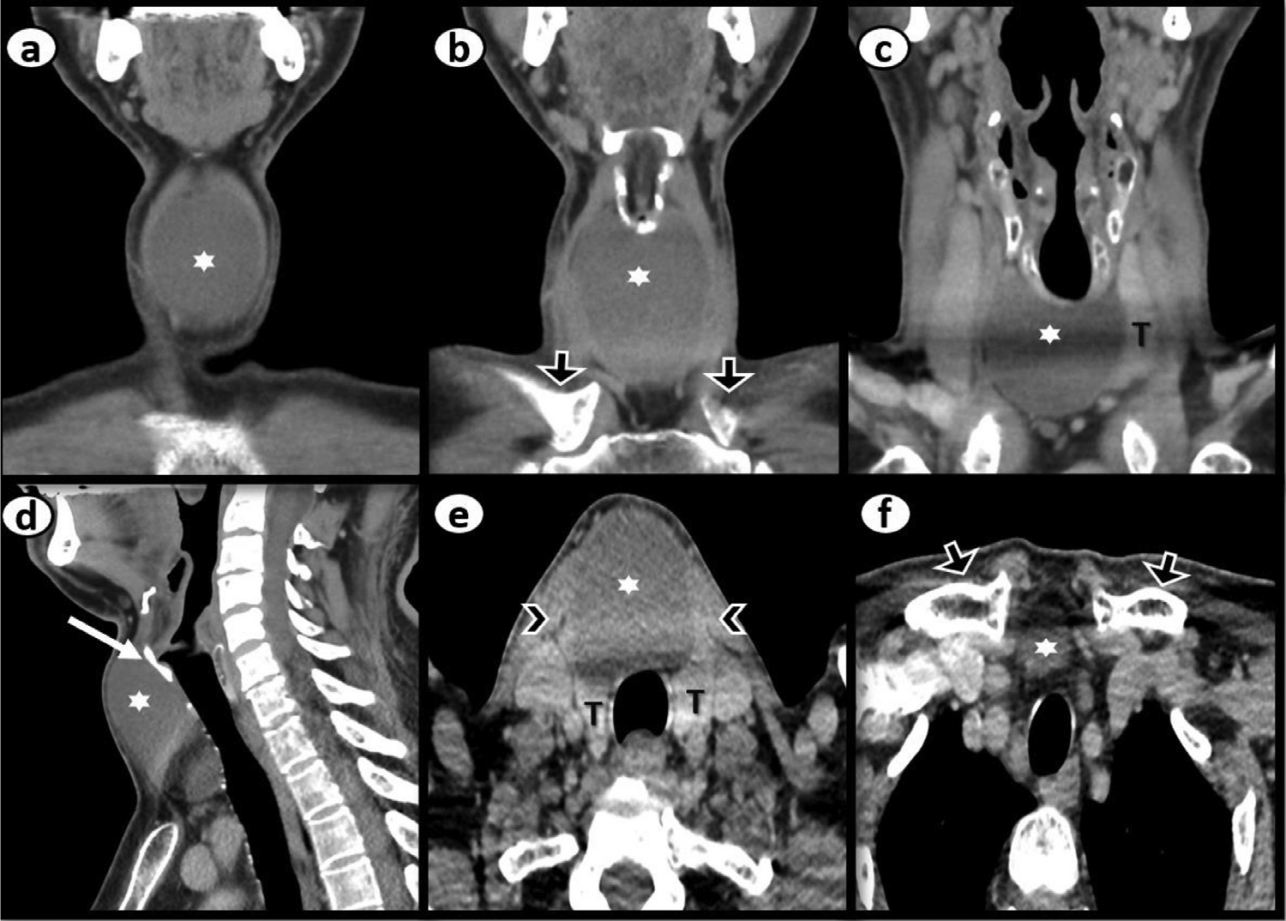
(a-f): Contrast enhanced computed tomography (CECT) neck: Coronal images showing colloid cyst (asterisks) from anterior to posterior (a-c). Sagittal image (d) showing extent of the cyst from thyroid cartilage (arrow) to just above the clavicles (open arrows b,f). Axial image (e) showing relationship between cyst (asterisk) and thyroid gland (T), note the anterolateral displacement of strap muscles (arrowheads).

**Fig. 6:**
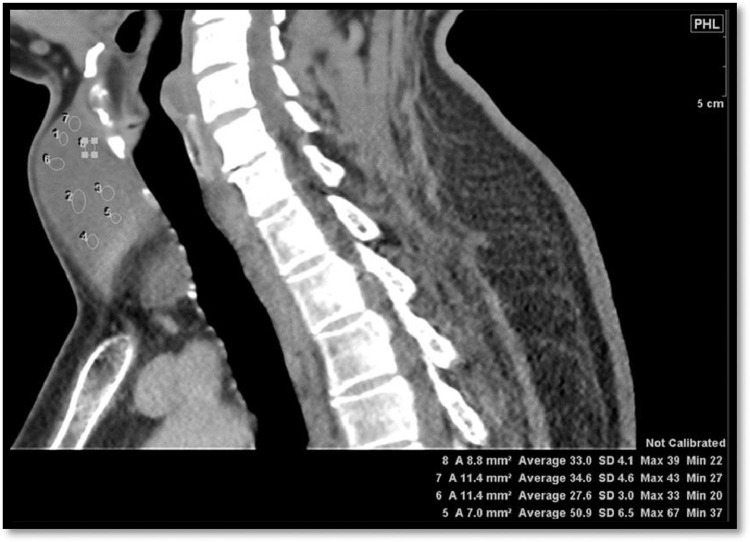
Sagittal CECT neck showing CT attenuation taken at different points of the colloid cyst (varied between 27-51 Hounsfield Unit.

**Fig. 7:**
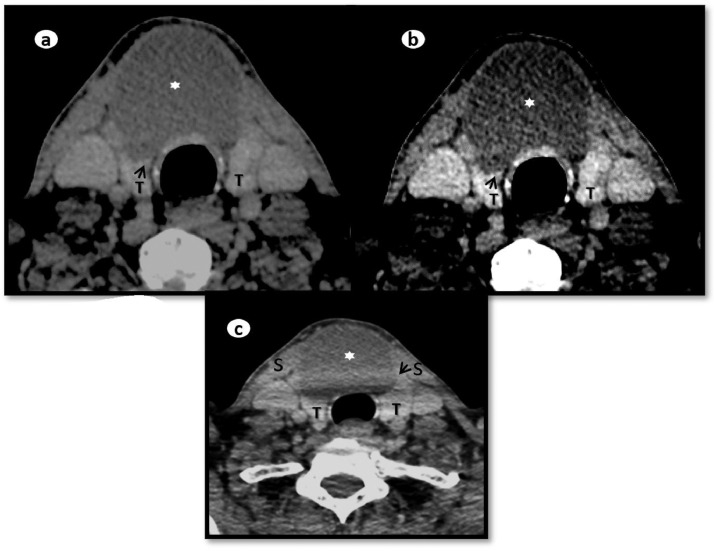
(a-c): Axial CECT neck showing colloid cyst (asterisks) showing “claw sign” in right lobe (a, arrow). Same axial section but with increased contrast for clear demonstration of the “claw sign” in right lobe (b). “Claw sign” in left lobe (c, arrow). Thyroid lobes (T) and strap muscles (S).

**Fig. 8:**
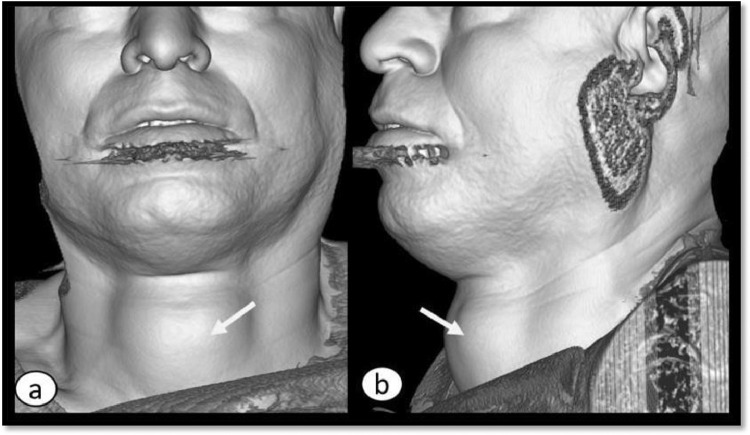
(a,b): 3D CT volume rendered images for skin, showing midline neck swelling (arrows).

**Fig. 9:**
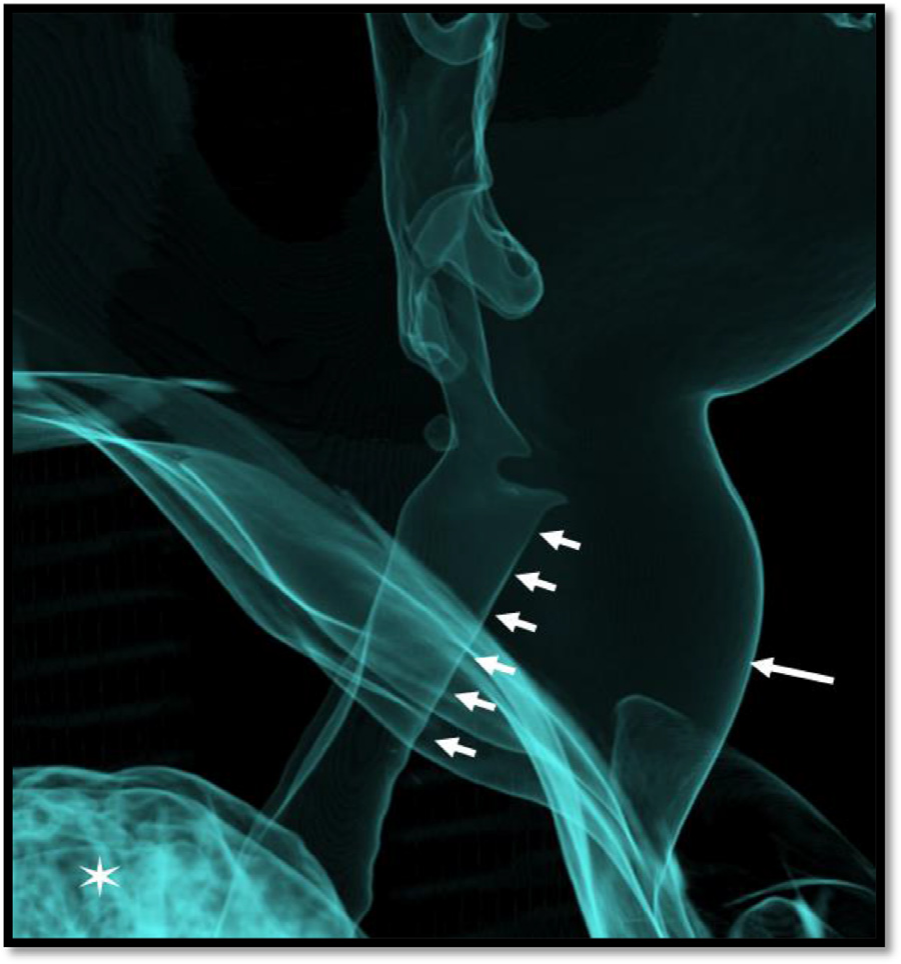
3D CT volume rendered images for air spaces showing no obvious compression effect by the colloid cyst on airway (multiple small arrows). The surface bulge of the neck swelling (long arrow). Air seen in the lung apices (asterisk).

Considering the differentials and ambiguous site of mass origin, referring team discussed the case with radiologist and planned to proceed for contrast enhanced computed tomography neck. Computed tomography (CT) showed a non-enhancing midline cyst in the region of isthmus measuring around 5.1 × 3.5 × 5.6 cm (transverse x anteroposterior x craniocaudal). It was pushing both lobes of the thyroid gland inferolaterally and strap muscles anterolaterally with no signs of aggression. The craniocaudal extension of the cyst was from the level of the thyroid cartilage to just above the clavicles (Fig. 5). The attenuation of the fluid content was varying between 27-51 Hounsfield Unit. No calcification was appreciated (Fig. 6). No worth noting mass effect or its connection with the airway was noted. On careful inspection of images, isthmus was unappreciable and we were able to see a “claw sign” in the thyroid lobes which helped us to discern the organ of origin (Fig. 7). The differentials of non-inflammatory thyroid cysts including atypical colloid and hemorrhagic cyst were given on CT scan with a suggestion of cyst aspiration to have definitive diagnosis. 3D volume rendered images for skin demonstrated clinically visible swelling (Fig. 8). 3D volume rendered airway images clearly showed no mass effect (Fig. 9). After observation of “claw sign” on CT scan we ruled out thyroglossal and branchial cysts from our differential list.

Lab results showed strongly positive antithyroid peroxidase antibodies (TPO), while thyroid stimulating hormone, triiodothyronine (T3) and thyroxine (T4) were normal. Rest of the blood examination findings were unremarkable.

Pathology assessment of the diagnostic aspirate showed, 18 ml of fluid having abundant colloid, few scattered foamy macrophages and hemosiderin. It was consistent with benign cystic colloid nodule with hemorrhagic changes, Bethesda Category II. It was found negative for malignancy. Fine needle aspiration (FNA) of the left thyroid nodule showed small groups as well as isolated follicular cells with focal Hurthle cell changes and rare collection of foamy macrophages in the background of thick colloid. Few clusters showed follicular epithelium with focal nuclear atypia including nuclear overlapping and grooving, putting it under Bethesda category III with further advise to have follow up and repeat FNA at appropriate interval.

Diagnostic aspiration was later followed by therapeutic colloid aspiration. On follow up visit, symptom of dysphagia was resolved. Due to atypical cells on histopathology and high-risk age group for thyroid malignancy, left hemithyroidectomy was done on subsequent visit. Patient did not report further complaints.

## Discussion

Colloid nodules are frequently formed nodules in a thyroid gland and can be either purely cystic or a part of solid thyroid nodule [2]. Sonographic detection of thyroid CC as an incidentaloma is not less common. Primary epithelial lined simple thyroid cysts are rare (2%-4%) but degenerative colloid nodules are seen frequently (82%) [7,8].

**Fig. 10:**
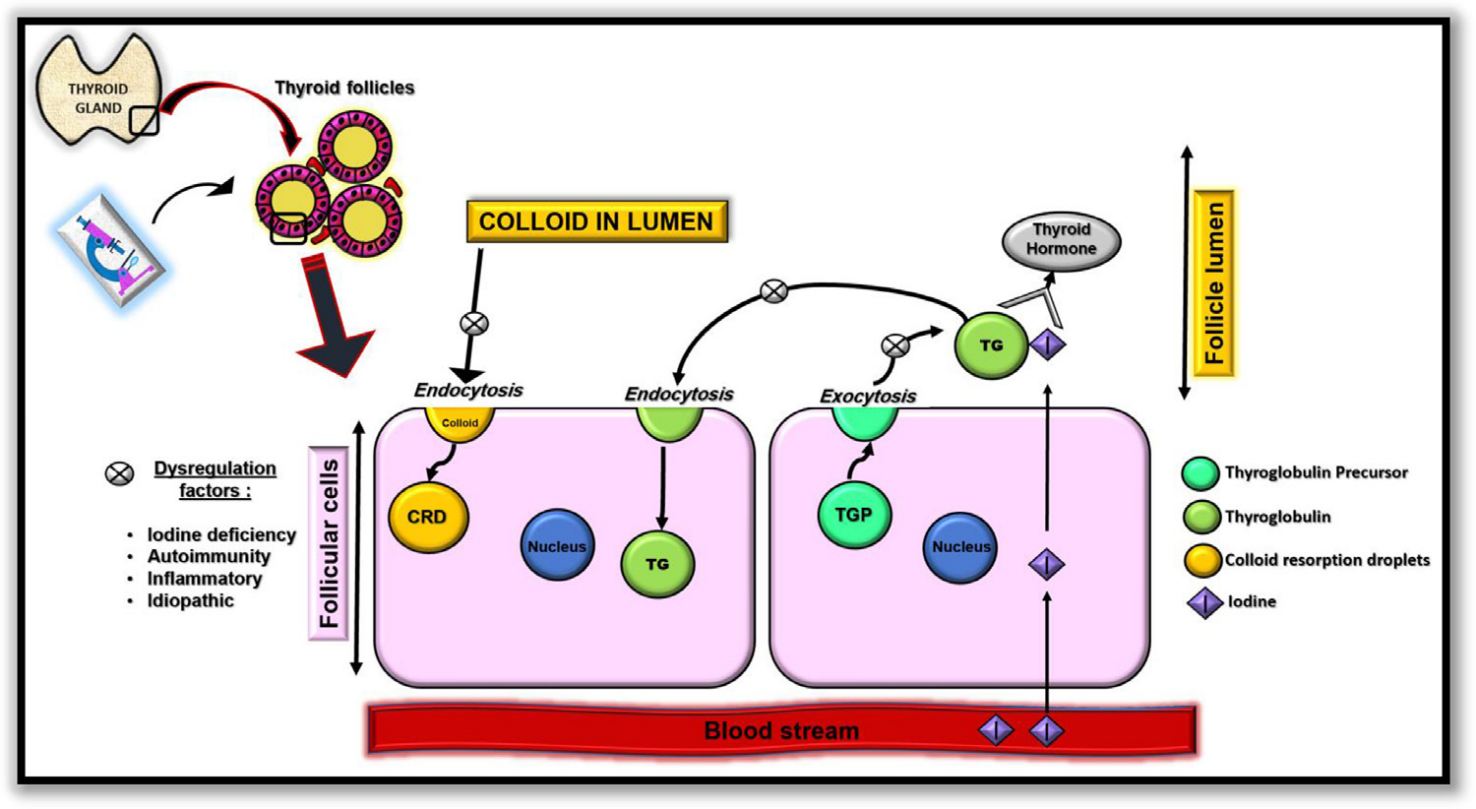
Simplified diagrammatic representation of proposed mechanism of formation of colloid nodule.

Its etiopathogenesis is still open for debate. In experimental studies CC was found rich in thyroglobulin, hence it is proposed to be formed as a result of destabilization in the physiological steps of thyroid hormone formation i.e. imbalance between endocytosis of a colloid droplet into the follicular cell and exocytosis of thyroglobulin into the colloid containing follicular lumen [6] (Fig. 10). It would not be inappropriate to state that CC are formed as a consequence of excess accumulation of colloid in thyroid follicle due to many proposed factors or causes. CC usually remains unnoticed by the patient unless it is enlarged and causes symptoms due to mass effect on airway, digestive tract or neurovascular bundle. More serious presentation can be neck pain due to internal hemorrhage or even its rupture which can be threatening. Spontaneous infection in the cyst is unexpected but any cyst can be infected if underwent needle aspiration [1,^7^,9].

Radiological imaging is fundamental in its diagnosis and ultrasound is usually adequate. Sonographically, anechoic thyroid cyst with multiple comet tail or reverberation artifacts are universally accepted to be a peculiar feature of CC indicating inspissated calcified colloid crystals and are considered diagnostic. If the cyst is very small having a single echogenic focus of colloid it gives cat's eye appearance. Literature has rarely mentioned some other appearances also like isoechoic or hypoechoic cyst, honeycomb pattern due to a large colloid clot and dependent avascular echogenic component due to debris or hemorrhage [2,^3^,^7^,^10^,11]. Hemorrhagic or debrinous changes seen at the base of cyst are believed to form a flat upper margin but our cyst had basal echogenic part with bulging convex upper border which was an added observation for us. Complete anechoic cyst without colloid crystals artifacts is not considered as CC but a simple thyroid cyst (which is epithelial lined and arises from pathological dilatation of any preexisting tubules, ducts or cavities formed during any stage of life). If these simple cysts become hemorrhagic or infected, they can mimic other uncommon appearances of CC [3,7].

If CC is enlarged more than 2 cm in size, it is at risk of intracystic hemorrhage due to increased intravenous pressure which could be spontaneous, after strenuous activity, external pressure or trauma [7,12].

**Fig. 11:**
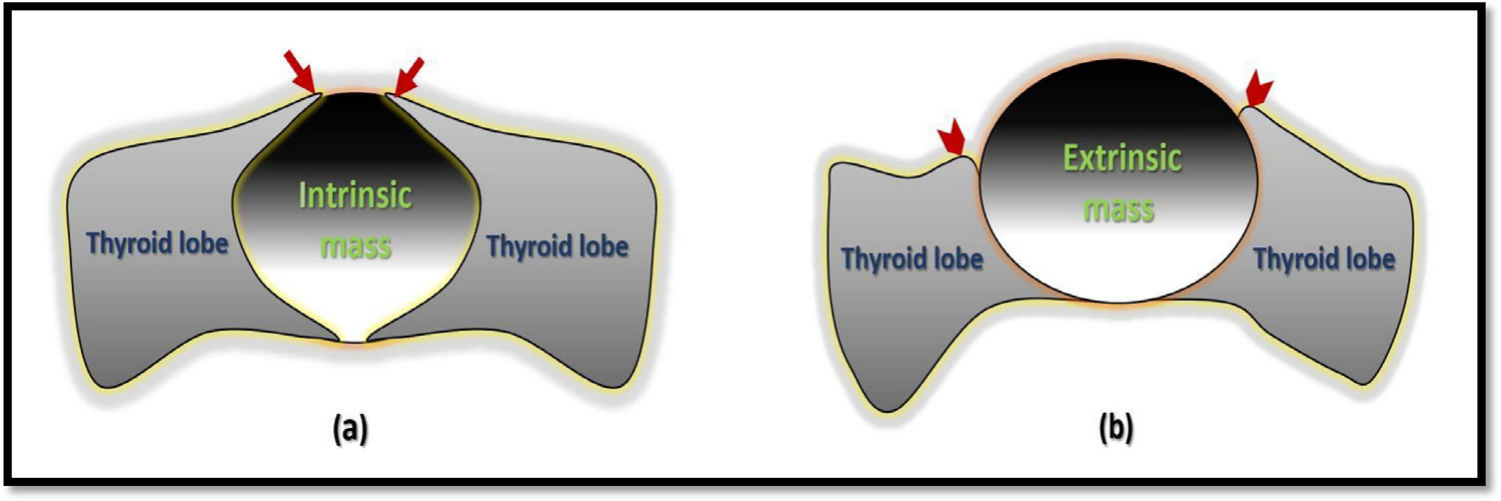
(a,b): Pictorial representation of difference between intrinsic and extrinsic thyroid isthmic mass. (a) Pointed claw like margins of thyroid tissue which partially wraps around an intrinsic mass (arrows). (b) Rounding of the thyroid margins (arrowheads) which does not wrap around an extrinsic lesion.

Cross sectional radiological examinations like CT or magnetic resonance imaging are not routinely required after ultrasound as they have less spatial resolution and lack of ability to describe the colloid crystals and varied cystic content. In doubtful cases, “claw sign” (beaked extension of organ solid tissue around the lesion) demonstration on these modalities can solve the dilemma. The claw sign is useful to determine whether a lesion is intrinsic or extrinsic, particularly in large lesions and small organs. It is more convincingly seen on cross-sectional imaging than ultrasound. The claw like appearance is formed when the mass originates within an organ parenchyma and grows towards its surface leading to thinning of partially surrounding spared parenchyma, which is in between mass and superficial surface of an organ. This parenchyma has sharp pointed ends unlike extrinsic mass which makes blunt parenchymal ends (Fig. 11).

Nuclear imaging is unjustified in cystic thyroid lesions [13], [14], [15]**–**16]. We opted CT scan for our patient because of unusual colloid appearance and undecided origin.

CCs are usually not much large but can be of variable size and found in any part of thyroid gland, though larger cysts have occasionally been observed in thyroid lobes [3,12]. Development of an oversized CC in isthmus over a period of only few weeks was an unexpected finding in our patient. Moreover, sonographic findings were also unanticipated for CC. The above attributes, to best of our knowledge have never been reported before.

Many societies have guided the clinicians and radiologist for management and categorization of cystic thyroid lesions. According to American College of Radiology Thyroid Imaging Reporting and Data System, CC comes under Thyroid Imaging Reporting and Data System 1 category of benign lesions, which do not need FNA [13,17].

As per recommendations, these cysts usually do not require even follow ups because they have no risk of malignancy and show no significant changes even on long term follow up. Only symptomatic benign cystic nodules can be simply aspirated both for diagnostic and therapeutic purposes, but recurrence rate is as high as 80% which is dependent on size and number of cysts. Post aspiration ethanol and radiofrequency ablation treatments of thyroid cysts are other options [1,^12^,^13^,18]. From histological view point, colloid nodule is under benign Bethesda category II (0%-3% risk of malignancy). It is subcategorized under benign follicular nodules which are defined as having predominantly colloid and follicular cells in variable amount. Microscopically, they are dilated follicles having flattened follicular cells lining with abundant central colloid. Other subcategories are hyperplastic or adenomatoid nodules, nodules in Grave's disease and macrofollicular subtype follicular adenoma [6,19]. CC are non-functional and have no effect on thyroid functions [7]. Despite benign outcome we believed that FNA was necessary in our patient as the cyst was large, superficial, symptomatic and had risk of rupture, hemorrhage or other complications.

Some possible differentials can be included according to the site of cyst, for example infrahyoid/intrathyroid thyroglossal cyst, intrathyroidal parathyroid cyst, branchial cleft cyst, intrathyroidal bronchogenic cyst and unilocular hydatid cyst. None of them were reported in the isthmus [20], [21]**–**22].

Incidental diagnosis of thyroid pathology is not uncommon. Thyroiditis and solitary thyroid nodule are amongst them. The risk of malignancy in solid nodules associated with thyroiditis is high [10,23]. We also came across these 2 pathologies in our patient. Autoimmune thyroiditis like Hashimoto's thyroiditis (HT) and Grave's disease (GD) can have similar sonographic demonstrations. There are some radiological features which favor oneone on other but do not negate anyone of them. Typical heterogenicity and variable texture abnormalities are seen more in HT [10]. Both HT and GD can have raised antithyroid peroxidase antibodies (TPO-Ab) but in GD 95% of patients have elevated anti-Thyroid stimulating hormone-receptor antibodies apart from TPO-Ab and anti-thyroglobulin antibodies. Tissue diagnosis is often not required for final diagnosis and the above findings are usually considered convincing for the management purpose [2,10]. We considered the diagnosis of HT in our patient because of positive TPO-Ab and micronodular heterogenous echotexture in a normal sized gland. The co-existence of CC and incidental thyroiditis in our patient cannot be correlated without any good explanation and were probably isolated pathologies which were diagnosed simultaneously.

Despite common thyroid nodule, colloid nodules can be confused with other differentials of neck cysts. Imaging modalities have the ability to recognize and diagnose atypical CC. It is important to diagnose it correctly to avoid exorbitant patient workup, long list of differentials, hospital admission and surgery.

## Patient consent

Informed consent was taken from the patient.
